# ABCA3 mutation-induced congenital pulmonary surfactant deficiency: A case report

**DOI:** 10.1097/MD.0000000000037622

**Published:** 2024-03-29

**Authors:** Chunxia Lei, Chunhui Wan, Caixia Liu

**Affiliations:** a Department of Neonatology, Wuhan Children’s Hospital (Wuhan Maternal and Child Healthcare Hospital), Tongji Medical College, Huazhong University of Science and Technology, Wuhan, Hubei, China; b Precision Medical Center, Wuhan Children’s Hospital (Wuhan Maternal and Child Healthcare Hospital), Tongji Medical College, Huazhong University of Science and Technology, Wuhan, Hubei, China; c Department of Pediatrics, Taihe Hospital, Hubei University of Medicine, Shiyan, Hubei, China.

**Keywords:** ABCA3 gene, congenital surfactant defects, inherited surfactant deficiency, neonatal respiratory distress, neonatal respiratory failure

## Abstract

**Introduction:**

Congenital surfactant deficiency, often caused by mutations in genes involved in surfactant biosynthesis such as ABCA3, presents a significant challenge in neonatal care due to its severe respiratory manifestations. This study aims to analyze the clinical data of a newborn male diagnosed with pulmonary surfactant metabolism dysfunction type 3 resulting from ABCA3 gene mutations to provide insights into the management of this condition.

**Patient concerns:**

A newly born male child aged 1 day and 3 hours was referred to our department due to poor crying and shortness of breath.

**Diagnosis:**

Primary diagnoses by the duty physicians were: neonatal pneumonia, neonatal respiratory failure, persistent neonatal pulmonary hypertension, birth asphyxia, myocardial damage, and arteriovenous catheterization. Genetic test revealed a compound heterozygous variant in the ABCA3 gene. One allele may be exon variant c.4561C>T, the second allele may be intron variant c.1896 + 2_1896 + 17del. The associated disease included pulmonary surfactant metabolism dysfunction type 3.

**Interventions:**

He was initially treated with an antiinfective therapeutic regimen.

**Outcomes:**

The family was informed of this condition and signed off, and the child died.

**Conclusion:**

Hereditary pulmonary surfactant deficiency is a rare and untreatable disease. The case highlights the challenges in managing congenital surfactant deficiencies and emphasizes the need for heightened awareness of this rare cause of infant respiratory failure.

## 1. Introduction

Surfactants, referred to as surface-active agents, often play a crucial role in the pulmonary region in reducing alveolar surface tension. These surfactants maintain a stable gas exchange space and prevent end-expiratory alveolar collapse. The congenital surfactant deficiency is favorable due to mutations in various genes that play essential roles in the biosynthesis of surfactants. The surfactant deficiency often leads to different clinical manifestations of severe respiratory distress syndrome with fatal respiratory failure in full-term neonates.^[1,2]^ Accordingly, the congenital surfactants deficiency is often associated with mutations in various genes, such as SFTPC, SFTPB, NKX2-1, or ABCA3, and CSF2RA, CSF2RB, SFTPA1, SFTPA2, SFTA3, as well as SFTPD, involved in the biosynthesis of surfactants.^[3,4]^ Among various genes, mutations in the ABCA3 double allele are the most common causes of congenital lung surfactant deficiency.^[5]^ In this report, we intend to analyze the clinical data of a child with pulmonary surfactant metabolism dysfunction type 3 due to ABCA3 gene mutation to explore the insights further. This case highlights the challenges in managing congenital surfactant deficiencies and emphasizes the need for heightened awareness of this rare cause of infant respiratory failure.

## 2. Case report

### 2.1. General information

Written informed consent was obtained from the legal guardians of the patient included in this study. The general information of the newly born male child included age of 1 day and 3 hours, gestational age of 40 weeks, G2P2, singleton, delivered vaginally, birth weight of 3.63 kg, very little amniotic fluid, as well as Apgar score of 7 points at 1 minute, 8 points at 5 minutes, and 9 points at 10 minutes. Despite being born at term with a normal Apgar score, the patient showed signs of respiratory compromise shortly after birth. The child was referred to our department due to poor crying and shortness of breath. Before admitting to our hospital, the child was intubated and ventilated at a local hospital. However, the treatment was ineffective, resulting in no improvement in the child with symptoms. The newborn male presented with poor crying and respiratory distress, necessitating admission to the hospital. The familial history included that the child’s mother had no history of diabetes mellitus, hypertension, and thyroid disease. Moreover, no specific family history of pulmonary-related diseases was reported.

### 2.2. Admission examination

The examinations during the admission were recorded as the temperature of 36.1°C, regular R50 beats/minutes, normal pulse of 140 beats/minutes, blood pressure of 73/42 mm Hg, transcutaneous oxygen saturation of 95%, drowsiness, poor response, mild trismus sign, coarse breath sounds in both lungs. Moreover, the heart rate of 140 beats/minutes, rhythmical, normal heart sounds, and no heart murmurs were recorded. The ventilator parameters were recorded as SIMV: FIO_2_ 60%, PIP 22 cm H_2_O, PEEP 5 cm H_2_O, and RR 45 times/minutes.

### 2.3. Ancillary tests

**Figure 1.**
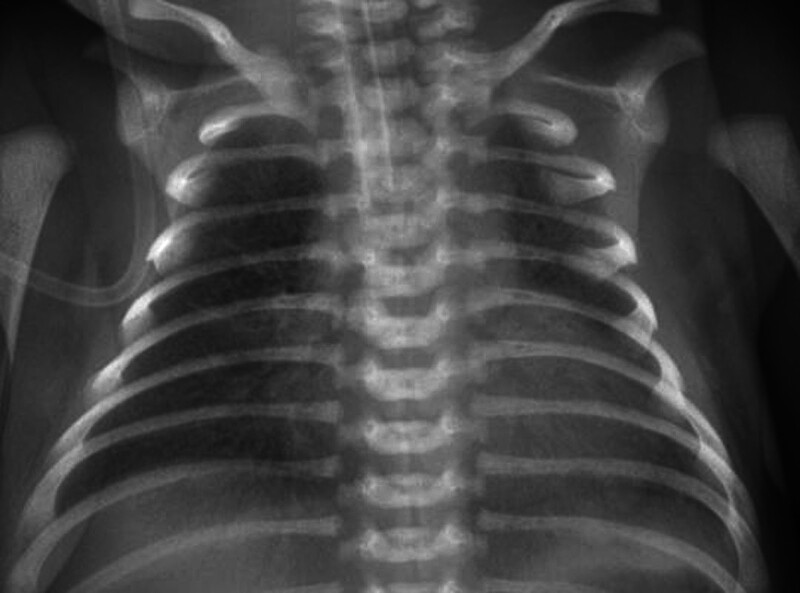
The admission chest X-ray examination shows a significant decrease in the translucency of both lungs, with “white lung”-like changes. The image shows the enhanced texture of both lungs, patches of faint shadows in both lungs, the whole heart shadow, and blurred diaphragmatic surfaces.

Various parameters of the blood gaseous analysis during the admission included pH of 7.47, PCO_2_ of 35 mm Hg, PO_2_ of 100 mm Hg, SO_2_ of 99.8%, HCO_3_ of 21.1 mmol/L, and SBE of 2.0 mmol/L, indicating respiratory acidosis and impaired gas exchange. Furthermore, the chest radiography suggested neonatal pneumonia (Fig. 1), characterized by patchy opacities and consolidations in the lung fields, confirming the presence of respiratory pathology. On the one hand, the bedside ECG suggested a first-degree AV block, right ventricular high voltage, and ST-segment changes, suggestive of myocardial injury and cardiac involvement. On the other hand, the bedside ultrasound suggested neonatal ductus arteriosus (trace). The abdominal ultrasound showed no abnormalities. The cranial ultrasound also showed no significant abnormalities. The complete blood picture analysis of quintuple classification (venous blood) included: L of 8.3%, N of 85.2 %, PLT of 163 × 10^9^/L, erythrocyte pressure volume of 44.0%, Hb of 152 g/L, RBC of 4.19 × 10^12^/L, WBC of 13.68 × 10^9^/L, elevated neutrophil ratio, and ultrasensitive CRP of 12.80 mg/L. In the case of myocardial damage, the serum myocardial enzyme profile (including AST) was determined, including creatine kinase isozyme-MB of 118 U/L, lactate dehydrogenase of 1156 U/L, lactate dehydrogenase isozyme-1 97 U/L, creatine kinase 2623 U/L. Notably, all these values were significantly elevated over the average values. Ancillary tests revealed abnormalities consistent with severe respiratory distress and myocardial damage.

### 2.4. Diagnosis and treatment

**Figure 2.**
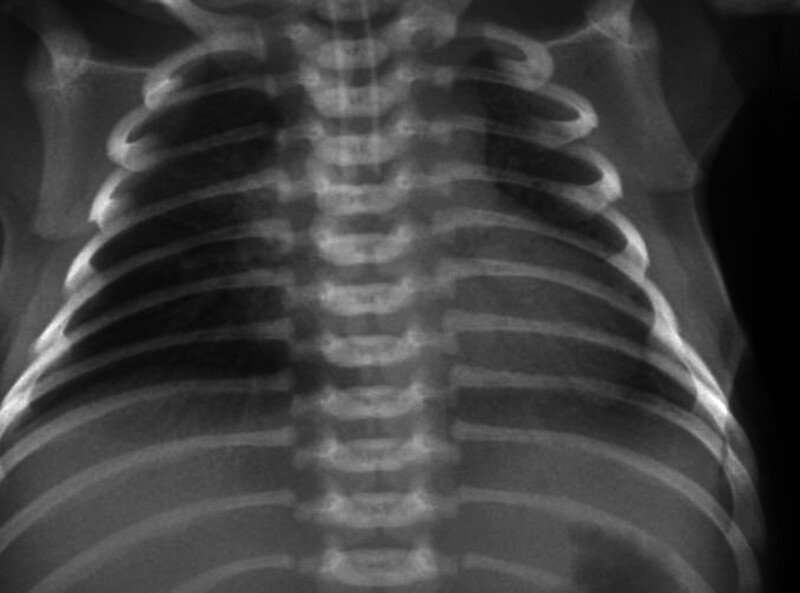
A chest X-ray review after intratracheal drip of lung surface-active substance suggests a slight increase in lung translucency on chest X-ray, proposing a slight improvement in lung perfusion.

Upon patient admission, the primary diagnoses by the duty physicians were: neonatal pneumonia, neonatal respiratory failure, persistent neonatal pulmonary hypertension, birth asphyxia, myocardial damage, and arteriovenous catheterization. Further, he was initially treated with an antiinfective therapeutic regimen. The ventilator parameters could not be adjusted downward. Additionally, the rechecked blood gas analysis included a pH value of 7.31, PCO_2_ of 57.90 mm Hg, PO_2_ of 61 mm Hg, SO_2_ of 88.0%, HCO_3_ of 28.5 mmol/L, SBE of 2.8 mmol/L, CO_2_ retention, poorer perfusion in both lungs than before (2021-04-28), and ARDS. Moreover, cardiac insufficiency and secondary neonatal respiratory distress syndrome were considered. The blood gas analysis was normal, and the ventilator parameters were gradually adjusted downward: SIMV: FIO_2_ 40%, PIP 20 cm H_2_O, PEEP 5 cm H_2_O, and RR 40 times/minutes. The lung translucency on the repeat chest radiograph was slightly higher than before admission, suggesting a slight improvement in lung perfusion (Fig. 2). During the treatment period, the lung ventilation status of the child was temporarily improved after repeatedly using endotracheal drops of exogenous pulmonary surface-active substance. However, the decrease in translucency was again apparent after about 24 hours. Considering the possibility of a genetic surfactant syntheses deficiency or metabolic disorders, such as SP-A and SP-B production deficiency, the genetic examination was conducted on 2021-05-21. It was observed from the results that a compound heterozygous variant of uncertain significance/possible pathogenicity was detected in the ABCA3 gene. The associated disease included pulmonary surfactant metabolism dysfunction type 3. Analysis of the genetic variants revealed potential disruptions in RNA splicing, resulting in altered protein-coding sequences and contributing to the pathophysiology of the disease. Eventually, the family was informed of this condition, and they signed off, and the child died.

## 3. Discussion

Respiratory distress often occurs in 5% to 7% of full-term neonates, manifested by shortness of breath, nasal flaring, intercostal or subcostal retraction, audible grunting, and purpling. In most cases, the neonates show mild and transient symptoms, often diagnosed as shortness of breath in the newborn. Other common reasons include persistent neonatal pulmonary hypertension, meconium aspiration syndrome, and infectious diseases, such as sepsis or pneumonia. Nevertheless, severe respiratory distress is typically caused by nonpulmonary modalities, such as congenital heart disease, air outflows, or anatomical abnormalities in the pulmonary airways. Intermittently, respiratory distress in term neonates is associated with inherited primary lung disease, such as surface-active substance deficiency.^[6,7]^

ABCA3 mutation-based deficiency is among the most common causes of hereditary surfactant deficiency in the pulmonary regions.^[8]^ This pathological condition has been considered in severe neonatal respiratory distress syndrome in cases of failed conventional treatments.^[9]^ The functional abnormality of the ABCA3 gene often leads to the rapid progression of neonatal distress syndrome, resulting in the death of the patient within the first 3 months of life.^[9]^ In our case, 2 variants of the ABCA3 gene were identified by sequencing the patient sample. It was observed that this variant could interfere with the RNA splicing, resulting in exon loss or altered intron and protein-coding sequences. Often, the identification of pure or compound heterozygous ABCA3 gene mutations usually resulted in more challenging clinical features and a poorer prognosis than patients with a single ABCA3 mutation than those without apparent genetic abnormalities,^[10]^ which were consistent with the severe profile and fatal outcome of the patient.

Currently, there is no profound approach for treating congenital surfactant deficiency. Nevertheless, Ciantelli et al^[11]^ described that ventilatory improvement of respiratory distress and utilization of steroidal antiinflammatory therapy was unhelpful in partially overcoming respiratory distress. Although the exact mechanism of this therapy remains unclear, improvements in respiratory function have been reported with azithromycin treatment.^[12]^ In several cases, improvements in patients with interstitial lung disease have been reported by applying hydroxychloroquine.^[13,14]^ However, it should be noted that no such therapeutic regimen was prescribed in this case. Notably, we observed a transient positive effect of improving the child’s symptoms and decreasing ventilator parameters after intratracheal drops of pulmonary surfactants. Considerably, the results were in agreement with the short-term results described in the literature.^[15]^

Further, several investigations are required to focus on individualized and precise treatment protocols for patients with ABCA3 deficiency. Although the confirmed diagnosis does not alter the prognosis of patients with congenital surfactant deficiency and is usually made after death, it is crucial to provide adequate counseling to the parents and family members of the neonates regarding the risk of recurrence. In summary, this article has summarized the clinical presentation and management of a child with congenital surfactant deficiency and its implications for understanding this disease. However, there were limitations. First, this report is limited by the lack of long-term follow-up data and detailed genetic characterization beyond the ABCA3 gene. Additionally, the retrospective nature of the study may limit the generalizability of findings and hinder the identification of potential confounding factors influencing the patient’s outcome. Furthermore, the study’s retrospective design limits the ability to assess the efficacy of specific treatment interventions and their impact on patient outcomes. Future prospective studies with larger sample sizes and comprehensive genetic analyses are warranted to address these limitations and provide a more comprehensive understanding of congenital surfactant deficiencies.

## Author contributions

**Conceptualization:** Caixia Liu.

**Data curation:** Chunxia Lei.

**Investigation:** Chunxia Lei.

**Methodology:** Chunhui Wan.

**Resources:** Chunhui Wan.

**Supervision:** Chunhui Wan.

**Validation:** Chunhui Wan.

**Writing – original draft:** Chunxia Lei.

**Writing – review & editing:** Caixia Liu.
